# Political Consequences of COVID-19 and Media Framing in South Korea

**DOI:** 10.3389/fpubh.2020.00425

**Published:** 2020-08-27

**Authors:** Wonkwang Jo, Dukjin Chang

**Affiliations:** ^1^The Institute for Social Data Science, POSTECH, Pohang, South Korea; ^2^Department of Sociology, Seoul National University, Seoul, South Korea

**Keywords:** COVID-19, framing, structural topic model, media, public health crisis

## Abstract

This study explored the Korean media's framing of COVID-19 and its impact on people's support for the government. A disaster such as a public health crisis has political consequences. COVID-19 is no exception. However, the direction of the effect is not easily determined. To properly understand this phenomenon, it is necessary to analyze how the media frames the crisis. Using Structural Topic Model, this study examines the Korean media's framing of COVID-19 and especially pays attention to international comparative framing. Based on our analysis results, we argue that expanded framing, which compared the quarantine performance of Korea and other countries, induced a positive change in people's attitudes toward the government, leading to a major political victory for the ruling party in the legislative election. Our research not only identifies the impact of international comparative framing on government support but also contributes to the development of methods for measuring media framing utilizing topic modeling methods.

## Introduction

Disasters have political consequences, and the COVID-19 pandemic is no exception. Under the pandemic, we can see an increase in the approval ratings of political leaders in several countries, including Italy, where rates of infection and deaths were exceptionally high. By contrast, the American president's approval rating remains steady, and Japan's prime minister faces a slight decrease in his approval rating (1). In South Korea, not only President Moon Jae In's approval rating increased under the COVID-19 pandemic, but also the increase led to a landslide victory of the ruling party in the legislative election. The result drew much attention from around the world because it was the first nationwide election held during the pandemic. The ruling Democratic Party earned 180 seats out of 300 the largest victory in the history of legislative elections in South Korea.

What makes the difference? When does a disaster induce an increase in a political leader's approval rating, and when does it diminish it? The most intuitive explanation is that political support is associated with government performance in protecting citizens from the disaster. This logic seems to explain the Japanese case, in which political support for the incumbent decreased as doubts grew about the government's ability to control COVID-19. However, this does not explain the Italian case, in which a rise in political support appeared, even though government performance in controlling COVID-19 was relatively weak. Another common explanation is the so-called “rallying ‘round the flag” effect (2), meaning that people rally around the government during crises such as war. This effect could explain the Italian case, but it does not explain the American and Japanese cases.

We suggest that more complicated and subtle political activities are at work, in addition to government performance. Among them, we focus on frame setting by the media. A disaster such as the COVID-19 pandemic has various aspects, and the parts the media choose to highlight, that is, how the media frame COVID-19, can determine people's responses to the threat. For example, if the media emphasize the role of the government and the need for national consensus in the COVID-19 crisis, rather than the objective performance of health policy, and if this approach earns public favor, the “rallying ‘round the flag” effect could emerge. On the other hand, if the media emphasize international comparisons of various countries’ disease control policy performance and present a vivid picture of a specific government's poor performance, the framing could induce a decrease in approval ratings. As framing theories and studies employing them have already claimed, both the actual threat and the way it is described are important (3, 4).

By exploring the COVID-19 framing features and changes in Korean media, we will analyze their impact on the Korean government's approval rating. There are two main reasons we selected the Korean case for analysis among many other countries. (1) In Korea, there was not only a rise in approval ratings but also a landslide victory for the ruling party in the legislative election. The analysis value is high because changes in approval ratings led to changes in the political power structure. (2) We pay particular attention to the international comparative frame of the Korean media and hypothesize that it would have had a great impact on the government's approval rating. As COVID-19 has demonstrated, the crisis caused by the virus crossed borders, meaning that international comparative framing could easily appear in other countries as well as in Korea. An analysis of the Korean case could contribute to the understanding of the political effects of international comparative framing in other countries.

The study's research questions are as follows:

(1) How was COVID-19 framed by the media in Korea?
(2) What is the feature of framing emphasizing international comparisons?
(3) How has this framing affected the Korean government's approval ratings?

## Materials and Methods

### Data Collection

We collected articles related to COVID-19 from 11 representative national daily newspapers in Korea. We used a database named “BigKinds” (https://www.bigkinds.or.kr/), which is run by the Korea Press Foundation and provides data on articles in Korea's leading 11 national daily newspapers. We collected all the items produced from January 20 to April 14, including the keyword “corona” in Korean (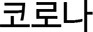). We used “corona” as our search term because it is the most natural and comprehensive word for referring to COVID-19 in Korea.

The reasons for establishing the period from January 20, 2020, to April 14, 2020, are as follows: On January 20, the first patient was confirmed to have been diagnosed with COVID-19 in Korea. From this point on, the framing of COVID-19 began in earnest. April 14 was the day before the legislative election in South Korea. Since the rise in support for the government was vividly revealed on the day of the election, we decided to examine public opinion and framing until just before the legislative election.

More specifically, we used a list of words in articles as data. BigKinds does not provide the original text of the item. Instead, it provides words that appear in each piece. For example, let us assume that a newspaper article's text body is as follows: “At today's meeting of the World…” In this case, BigKinds will produce a list of words in the article including “At, Today, Meeting, World…” This type of data is suitable for use in topic modeling methods such as the structural topic model (STM), which we used. We excluded duplicate articles from the initial search results. Newspaper companies sometimes republish the same article with only slight changes to a word or phrase, but such duplicate items were unnecessary in our analysis. We therefore removed articles that BigKinds categorized as repeated articles. However, because BigKind's information is incomplete, we checked whether the first and last 50 characters in the list of words for each article were the same. If we found duplication in either part, we removed the item. Finally, 37,184 articles were used as data.

## Methods

Our main method was STM, a type of topic modeling method, which, in addition to the essential function of such methods to estimate multiple topics from large quantities of documents, estimates changes in the proportion or content of the estimated topics according to the meta-information of the documents (5, 6). Meta-information in a document refers to various information belonging to the document other than the content of the document, e.g., document publication time, document category. We utilized the publication time and type of newspaper as key meta-information to estimate the changes in the proportions of topics statistically. Estimating topics from 37,184 articles with STM makes it possible to analyze main subjects or framings more objectively and efficiently.

The process of estimating multiple topics from a large number of documents using topic modeling methods is based on several assumptions. Most of the topic modeling methods developed after latent Dirichlet allocation (LDA), the most commonly used topic modeling method (7), share these assumptions and processes. First, a document is assumed to be a “bag of words,” and only information about the frequency and type of each word is utilized, not the actual sentence. This assumption is the reason we can fit our model with word list information from each newspaper article in BigKinds without the original text. Second, a topic is assumed to be a probability distribution of words. In this probability distribution, a word that is important for a topic has a high probability, while a word that is not has a low probability. This assumption is reasonable, given that a topic is realized in language material through an unequal use of words. For example, the topic “Banning foreigners from entering the country” can be written as a probability distribution of words such as [foreigners−0.02, entry−0.01, prohibition−0.01, border−0.009…]. Third, it is assumed that each document is generated from multiple topics and a probability distribution of the topics unique to each document. The distribution of topics held by a document refers to the proportion of multiple topics in that document. For example, if three topics were estimated in the entire document, one document could have a topic distribution such as [Topic 1–0.4, Topic 2–0.4, Topic 3–0.2]. This is also a reasonable assumption, given that a single document—in this case, a newspaper article—can have a variety of topics simultaneously.

Topic modeling methods after LDA, including STM, estimate topics (i.e., probability distributions of words) and document-specific topic distributions that are most likely to generate given documents (8, 9). Extracting topics from documents has become a statistical problem, as documents are viewed as bags of words, and the topic is assumed to be a probability distribution of words. Specific estimation algorithms vary, and algorithms such as Gibbs sampling and variational inference are the best known (8, 10).

We propose that topics from topic modeling methods are valuable for analyzing framing, which is why we utilized STM. According to framing theory, the critical features of framing are selection and salience. In other words, selecting specific aspects and making them salient is framing (3, 11, 12). If we pay attention to a set of words with a high probability from an estimated topic, we can deduce the most salient objects in the given data because it is natural that important and salient objects are frequently referred to. Moreover, a set of words with a high probability also provides information on the context of important objects. It is difficult to understand the meaning and value of an object from just one word referring to the object. However, if we have a set of high probability words, we will be able to infer the context in which they are used, and we can more accurately estimate the nature of the objects. For example, when the word “Japan” has a high probability, it is difficult to know exactly what it means. However, if words such as “colonial land,” “trade,” “conflict,” “Korea,” and “revenge” also have a high probability, the meaning and value of the object “Japan” is more apparent in the data. In a nutshell, a topic, or probability distribution of words, is valuable information for analyzing framing because we can identify essential objects and their context.

The specifics of our STM and additional data preprocessing are as follows. We assumed that the publication time of an article and the newspaper category of an article based on political perspectives could affect the proportion of the topics estimated by STM in the documents. The unit of a day measures the publication time of items, and one of the three values of the newspaper category is assigned to each newspaper. The names of the newspapers and the newspaper category of each are shown in Table 1. If topics from STM provide information on framing, changes in the proportion of topics according to other variables provide clues on changes in framing, depending on the variable.

**Table 1:** Newspapers and their categories.

**Newspaper name**	**Category**
Chosun-ilbo	Conservative
Joongang-ilbo	Conservative
Donga-ilbo	Conservative
Hankyoreh	Liberal
Kyunghyang Shinmun	Liberal
Kookmin-ilbo	Other
Naeil Shinmun	Other
Munhwa-ilbo	Other

In our STM, we used only the words that appeared in five or more articles. Terms used in only a small number of items do not provide suitable information for estimating topics. Finally, 45,905 different words were used in our model. Note that the term “
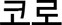
”(in English “coro”), which BigKinds incorrectly extracted from sentences, was corrected as “
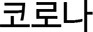
” (in English “corona”).

We also used Walktrap, a network community detection algorithm for categorizing topics into cohesive subgroups (13, 14). STM also provides information on the correlation among estimated topics. A positive correlation between two topics indicates that the two topics tend to appear in the same document. We assumed that a positive correlation between two topics represented a link between the two topics. Based on this assumption, we built a network among the estimated topics and applied Walktrap to detect relatively cohesive communities of topics. Finding cohesive communities of topics allowed us to infer larger subjects embracing individual topics, which are also valuable information for analyzing framing. Communities of topics present not only specific important objects but also common features of important objects at a more abstract level.

There are many types of network community detection algorithms. We chose Walktrap because it is resilient to the “resolution problem,” which refers to the incapability of detecting a community consisting of a small number of nodes, and its strong performance in several experiments (13, 15). We set the Walktrap step parameter to 2, meaning that it calculated the distance between nodes based on a two-step random walk.

All the analyses explained above were performed using R (16) and its packages, including the following: “tidyverse” (17) (for data wrangling and visualization), “tidytext” (18) (for data wrangling), “tidyr” (19) (for data wrangling), “stm” (5) (for STM), “igraph” (20) (for network analysis), “widyr” (21) (for data wrangling), “lubridate” (22) (for handling date), “ggrepel” (23) (for visualization), and “ggraph” (24) (for visualization).

## Results

We estimated 80 topics from 37,184 newspaper articles. As previously explained, a topic is a probability distribution of words (in this case, 45,905 words) and does not have an intuitive meaning. Topics need to be interpreted by human researchers. We interpreted each topic based on three types of information: 20 words with the highest probability in each topic, 20 words with the highest frequency-exclusivity (FREX) score, and 10 documents' titles that contained the highest proportion of each topic. The high probability words indicate the essential objects in the topic. The FREX score supplements the probability. Suppose a word has a high probability for all topics. The word paradoxically does not contain useful information about individual topics. The FREX score is an indicator to overcome this by considering the exclusivity and frequency together (6); that is, a high FREX score word for a topic is important, especially within the topic. Documents with a high proportion of a topic present the realization of the topic in language materials. Two authors considered the three types of information and labeled 80 topics based on an agreement. The results are shown in Table 2. The first column contains topic numbers (a nominal value for distinction), and the second column contains labels assigned by the authors. The third column contains numbers assigned to cohesive communities of topics detected by Walktrap. This number is also a nominal value for the distinction between communities of topics. As explained in the Methods section, the communities of topics are used to identify larger subjects or themes from related topics.

**Table 2:** Topics and topic communities.

**Topic #**	**Interpretation**	**C#**
6	Museum events (e.g., online exhibitions)	1
9	COVID-19-related gossip of celebrities	1
10	COVID-19 and fine dust or atmospheric conditions	1
14	Rent reduction campaign to overcome COVID-19	1
19	Donations to overcome COVID-19	1
22	Interruption of church services	1
26	Disputes concerning event and travel cancellation penalties and related government policy	1
29	News on farmers and sales of agricultural products during the COVID-19 crisis	1
32	Prosecutors' investigations and court rulings	1
33	News on various broadcast programs and shows	1
34	Issues related to mask supply (prevention of hoarding, supply increase, etc.)	1
36	Dramatic stories of families that occurred because of COVID-19	1
39	Political conflict over various remarks, including hate speech related to COVID-19	1
40	News presenting a quiet street scene	1
43	COVID-19-related conferences with the president	1
44	Online consumption growth and distribution industry	1
46	News relating to prayer or sermons	1
48	Introduction of various self-defense methods focusing on disinfection methods	1
62	Film and film industries	1
69	Closure of various facilities for quarantine	1
70	News about how to relieve depression or anxiety caused by COVID-19	1
72	News of investigations into false information and rumor dissemination	1
79	Reviews of society and the world written in consideration of COVID-19	1
5	The effect of COVID-19 on the air transport industry	2
16	Suspension of factory operations due to COVID-19 (Hyundai Motor, etc.)	2
20	Changes in the working form of companies due to COVID-19 such as telecommuting	2
25	Changes in economic indicators such as exports due to COVID-19	2
30	News on economic prospects	2
42	Government regulations relating to employment and labor-related issues arising from COVID-19	2
50	The financial performance of major companies and their stock price prospects	2
52	Development of mobile application related to COVID-19 and support for the development	2
58	Lack of blood supply due to COVID-19 and group blood donation	2
61	New vehicle launches and sales situation	2
65	News on the stock market	2
71	News on the government's extra budget	2
75	News on major companies (stories of CEOs, etc.)	2
76	Policies to support small business owners and small- and medium-sized enterprises affected by COVID-19	2
78	Financial support policies for companies such as loan support	2
4	Dr. Li Wenliang's death and President Xi Jinping's weakening political base	3
7	COVID-19-related news on major cities in China such as Wuhan	3
17	Events and conflicts related to quarantine facilities for Korean residents in Wuhan	3
21	COVID-19 in the United States and the U.S. Government's response	3
24	The novel coronavirus generation process and infection path (including a description of the Chinese region)	3
28	U.S. political news	3
37	COVID-19 confirmed patient statistics (many news reports from China)	3
38	Efforts to develop vaccines and treatments for COVID-19	3
45	COVID-19 reaction of U.S. forces in Korea and South Korean forces	3
49	COVID-19-related situations in Italy and other European countries	3
51	News on bans to entering countries	3
54	COVID-19 infection of political leaders in other countries	3
56	News on North Korea	3
59	News on games	3
60	COVID-19 patient occurrence news	3
63	International relations and diplomatic news	3
64	COVID-19 collective infection of Japanese cruise ship passengers	3
73	News on Japan (e.g., Prime Minister Abe)	3
27	Postponement of school openings, academic schedule adjustments, and other related issues	4
47	Colleges' COVID-19 reactions, including the postponement of the opening of classes	4
77	News of the closure of childcare institutions and private education institutions	4
23	Deferring and canceling major sporting events, including the Tokyo Olympics	5
35	Golf tournament news	5
57	Sports news	5
1	News about the Sincheonji church	6
2	COVID-19 news from Daegu	6
3	Symptoms and numbers of confirmed or infected patients	6
8	Various policies to prevent infection (e.g., social distancing)	6
12	News of deaths from COVID-19	6
15	Collective infection cases and patient news (care centers, Zumba dance academies, PC rooms)	6
31	Confirmed patients' contact tracing	6
41	Responses and activities of various government ministries and local governments with respect to quarantine	6
53	The hospital and medical staff situation (lack of beds, the fatigue of medical staff)	6
55	News of domestic patients or confirmed patients	6
66	COVID-19-related news on Jeju	6
67	COVID-19 test results (including those of key politicians and other important people)	6
68	News on confirmed patients from various locations	6
74	Various measures to prevent collective infection at work (such as measures to prevent infection of call-center staff)	6
11	News of the April 15 legislative election	7
13	News of each political party (mainly on the legislative election)	7
80	Issues of voting in general elections such as pre-voting	7
18	Prices of apartments and real estate falling due to COVID-19	8

**Figure 1:**
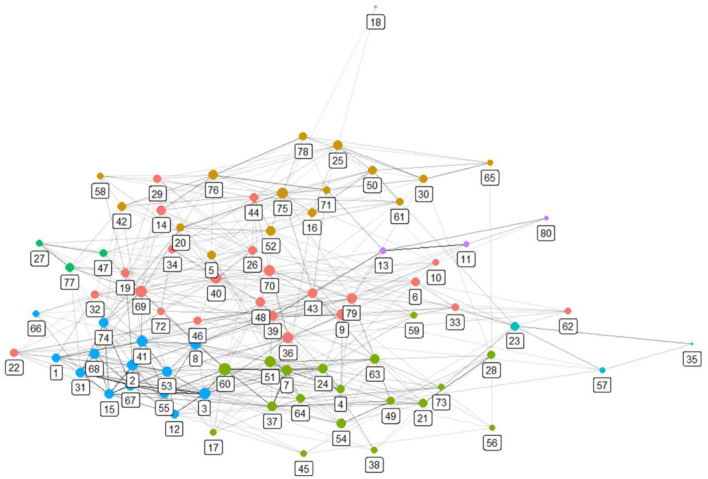
Topic network.

Though extracting 80 topics from 37,184 documents produces an excellent summary, the 80 topics represent, nevertheless, a lot of information for humans to grasp intuitively. As previously mentioned, we formed a network of the topics and identified whether there were cohesive communities among them. Figure 1 presents a visualization of the network. Each node represents a topic, and nodes of the same color belong to the same community. Refer to the second and third columns of Table 1 for topics belonging to each topic community. As previously mentioned, each topic community is numbered, and this is the third column of Table 2. In Table 2, 80 topics are arranged by the topic community number. We interpreted topic communities in consideration of topics belonging to each topic community. In other words, a more abstract subject to describe each topic community was derived in consideration of its topics. The result is Table 3.

**Table 3:** Topic communities' interpretation.

**Community #**	**Interpretation**
1	Impact of COVID-19 on everyday life and response of government and citizens
2	Impact of COVID-19 on economy and markets and response of businesses and government
3	COVID-19 situation in other countries and international relations
4	News of schools and other educational institutions and their COVID-19 response
5	Sports news
6	News of confirmed patients, medical staff, and major infection clusters
7	News on the legislative election
8	Falling real estate and apartment prices

**Figure 2:**
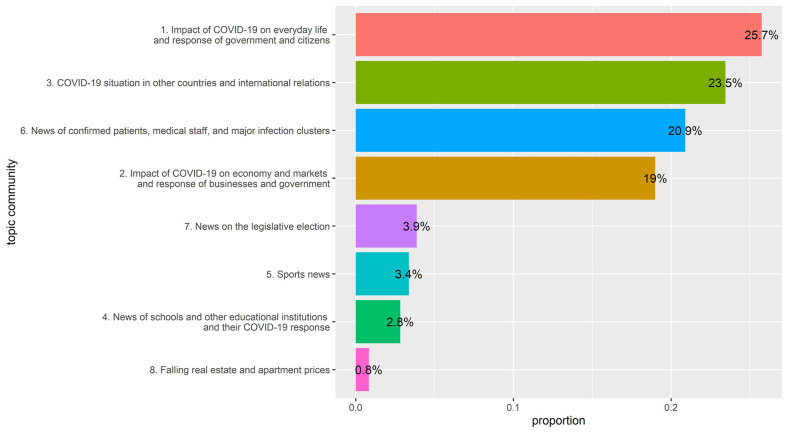
Proportion of topic communities.

STM allows us to estimate the proportion of each topic in the entire document. By adding the proportions of topics in the same topic community, the proportion of each topic community can also be estimated. Figure 2 shows the results.

While it was predictable that topic community No. 1 dealing with everyday life and No. 6 delivering medical information would be high, the fact that No. 3 had the second highest proportion is a notable result. This result reflects the fact that COVID-19 is a global crisis. We looked at the change in the proportion of topics in the third community, depending on time and newspaper type. We selected topics 4, 7, 21, 49, 51, 54, 64, and 73, which are significantly related to news of other countries, from topic community no. 3 to estimate their proportion changes. Furthermore, we visualized the change in President Moon Jae In's approval rating over the same period to see if the proportion trends of these topics were similar to that of the president's approval rating. Approval rating data is derived from the Gallop Korea Report (25–30).

**Figure 3:**
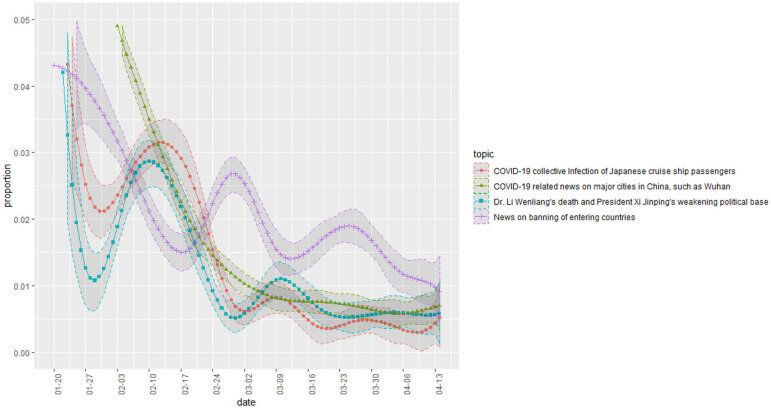
Topic proportion change (1).

**Figure 4:**
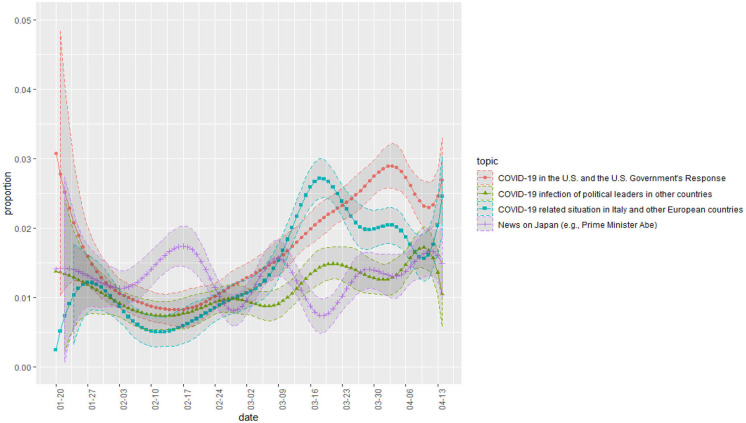
Topic proportion change (2).

**Figure 5:**
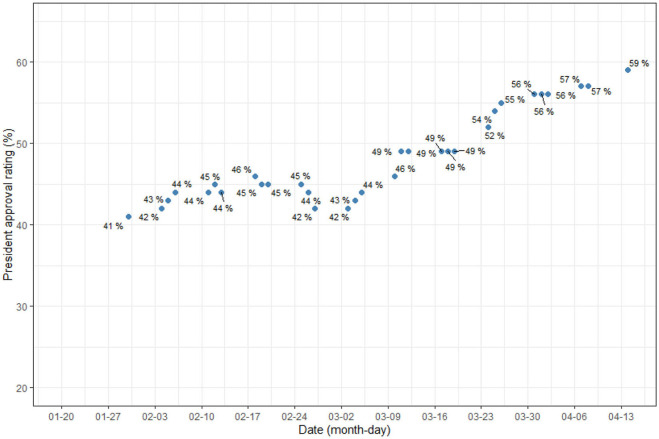
President's approval rating.

Figure 3 is a plot of the change in proportion over time in topics whose proportion decreased over time. The topics related to China, entry bans, and Japanese cruise ships. Figure 4 shows some topics that increase slightly or maintain a steady proportion. They are topics on foreign countries (relative to Korea) other than China. Figure 5 shows a change in the presidential approval rating, representing an increase over the same period. The topic proportion change in Figure 4 and the change in presidential approval rating are more similar. Table 4 shows the correlation coefficients between the presidential approval rating and the topic proportion in Figure 4.

**Table 4:** Pearson correlation coefficients between topics in Figure 4 and approval rating.

	**Pearson correlation coefficient**	***p***
Approval rating and Topic 21 (COVID-19 in the United States and the U.S. Government's response)	0.9161648	4.868e−13
Approval rating and Topic 49 (COVID-19-related situations in Italy and other European countries)	0.71208	7.035e−06
Approval rating and Topic 54 (COVID-19 infection of political leaders in other countries)	0.7439068	1.617e−06
Approval rating and Topic 73 (news on Japan)	0.1738349	0.3497

These correlation coefficients have limitations. Since the approval rating of the president was not measured every day, the approval rating over 31 days and the proportion of topics corresponding to those days were utilized: from January 20 to April 14, a total of 86 days, there are only 31 data points. However, the results show that the three international comparative topics (No. 21, 49, 54) have significant positive correlations with the approval rating.

**Figure 6:**
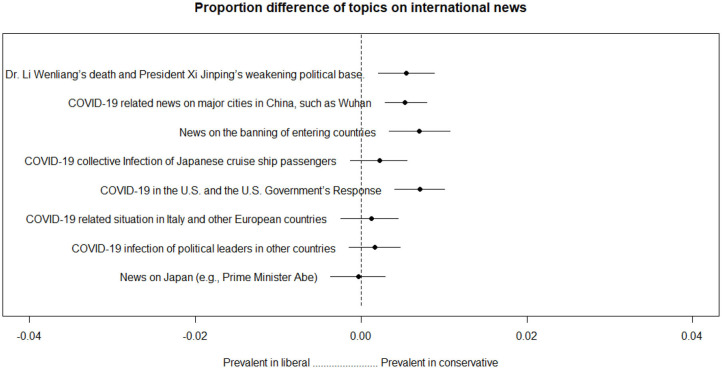
Proportion differences among topics concerning international news.

Additionally, we estimated how the above eight topics differed in proportion, depending on the type of newspaper. Specifically, we looked at the difference in proportion between liberal and conservative newspapers. The results are shown in Figure 6.

Topics concerning China, the United States, and the ban on entering countries are more prevalent in conservative newspapers than in liberal newspapers.

## Discussion

The South Korean government experienced a dramatic rise in its approval rating during the COVID-19 crisis. According to Gallop Korea, the percentage of those who stated, in the performance assessment, that the president was “doing well” rose to 59% during the April 13–14 period, just before the legislative election, from 46% during the January 14–16 period, just before the first confirmed case of COVID-19 occurred. A total of 54% of respondents who gave positive reviews April 13–14 cited the government's strategy in coping with COVID-19 as the reason. Based on this increased approval rating, the ruling party achieved an overwhelming victory in the legislative election, which was unprecedented in South Korea's political history. It won 180 out of a total of 300 seats.

With 10,506 confirmed cases and 222 deaths by April 14, how could the South Korean government and the ruling party have been able to win such generous support? We think that news about other countries and the international comparative framing that resulted can at least partly explain this. In our view, there were two crucial framings related to news of other countries and their changes.

The first was the attempt and decline of the Chinese entry ban framing. By the time South Korea had started to experience COVID-19, China was in the middle of a disease epidemic. During this period, media focused on news of China's infections and the need to ban Chinese entry into South Korea. The high proportion of topics related to Chinese news and entry issues in the early stage (Figure 3) shows an active attempt to establish such a framing. Given that such topics had a higher proportion in conservative media (Figure 5), it is likely that it was mainly conservative newspapers that attempted this. However, as shown in Figure 3, this framing was not well received by the public and gradually declined in proportion.

Instead, the emerging framing emphasized the excellence of Korea's quarantine performance based on international comparisons. As Figure 4 shows, as COVID-19 spread worldwide, news of infections in the United States and European countries started to take on greater proportions in reports. The infection status in these countries was much worse than in Korea. As these topics expanded their weight, we believe that the conditions formed for a change in domestic public opinion in favor of the current administration. In other words, as international comparisons became more active, Korea's performance was confirmed to be relatively superior, which had a positive impact on the government's approval rating. We speculate that this is the reason the change in the proportion of topics in Figure 4 and the change in the presidential approval rating of Figure 5 seem similar.

In sum, we propose that the following occurred. When COVID-19 broke out in South Korea, the legislative election was expected in less than three months' time. The conservative newspapers, well known for their very critical stance toward the current liberal government, seemed to want to use the pandemic to make the ruling party lose. They repeatedly reported negative news. The major logic of their criticism was that South Korea did not ban Chinese entry because the current liberal government was shamelessly subservient to China due to its pro-North Korea policies. Some conservative media even argued that the South Korean government was even more incompetent than the North Korean government because the latter banned Chinese entry very early as COVID-19 broke out. This frame-setting left a strong impression in voters' minds that the government's performance in fighting COVID-19 was the most important criterion when casting their votes. Then, there was a turnaround. While South Korea succeeded in flattening the curve, regions that were considered to be more advanced such as the United States, Japan, and Western Europe began to suffer. South Korea became a model of best practices in the world, in terms of quarantine. The impression that government performance was the most important criterion for casting votes in the legislative election still existed. Voters cast their votes according to this formula, and the landslide victory of the ruling party followed.

A public health crisis is an event in which people see the government's capacity clearly, so it has numerous triggers that can change people's attitudes toward the government. Support for the government has a tremendous impact on the resolution of such a situation. The results of this study show that consideration of framing is necessary to accurately predict changes in government support during these crises.

This study also contributes to the development of methods for measuring media framing. Measuring framing is difficult because data are vast, and a small number of researchers cannot review it all. This study presents the possibility of analyzing framing through computer and human collaboration. While the topics derived from topic modeling methods are not the equivalent of framing, they can provide adequate information regarding framing. Researchers can measure framing efficiently and accurately by making good use of such methods. During an outbreak of a population-wide infectious disease, the news framing government policy can greatly influence disease trends by affecting public opinion. However, measuring framing quickly and properly responding to it is difficult. Our approach using topic modeling will contribute to the formulation of efficient public health policies considering media framing.

The main limitation of this research is that our analysis was limited to traditional media: the major newspapers. As is well-known, a variety of new media such as YouTube and various social media have recently emerged. Future analyses should also cover the framing of these media. Another limitation is that a more sophisticated time-series analysis could not be attempted. This study analyzed only the simple correlation between the approval rating and the topics' proportion because of the limitations of the data. It is necessary to obtain more data in the future and attempt sophisticated time-series analyses such as cross-correlation analysis.

## Data Availability Statement

Publicly available datasets were analyzed in this study. This data can be found here: https://www.bigkinds.or.kr.

## Author Contributions

WJ: data analysis, result interpretation, and writing manuscript. DC: research direction setting, result interpretation, and writing manuscript.

## Conflict of Interest

The authors declare that the research was conducted in the absence of any commercial or financial relationships that could be construed as a potential conflict of interest.
